# Hidden-break diversity in pancrustacean rRNA profiles

**DOI:** 10.7717/peerj.20693

**Published:** 2026-02-03

**Authors:** Aitana Casanova Gómez, Francesc Mesquita-Joanes, Ferran Palero

**Affiliations:** Cavanilles Institute of Biodiversity and Evolutionary Biology, Universidad de Valencia, Paterna, Spain

**Keywords:** 28S rRNA, Hidden break, Pancrustacea, Oligostraca, Branchiopoda, Malacostraca, Fragment analyzer, D7a, rRNA secondary structure

## Abstract

**Background:**

In the 28S rRNA molecule of many invertebrates, a hidden break splits this large subunit into two noncovalently associated fragments (28Sa and 28Sb), masking 28S in electrophoretic profiles and biasing the standard measurements of RNA quality in extracted tissue samples. Pancrustacean diversity in RNA hidden breaks remains incompletely surveyed, particularly for Oligostraca.

**Methods:**

We sampled 12 species spanning Branchiopoda, Malacostraca, and Oligostraca around Valencia (Spain). RNA was stabilized with DNA/RNA Shield, extracted with Quick-RNA MagBead, and profiled on an Agilent 5200 Fragment Analyzer. Peaks were assigned to 18S and 28S fragments using BLAST-inferred gene lengths from reference genomes and annotated rDNA. We analyzed 28S secondary-structure domains (D-regions) using RNAfold and focusing on D3 and D7a.

**Results:**

Oligostracans and most branchiopods analyzed showed the canonical single-peak profile consistent with 18S, 28Sa, and 28Sb of similar size. Malacostracans exhibited greater profile diversity, including multiple distinct peaks attributable to expansions that alter the relative sizes of 28Sa and b, including expansions near D7a. Comparative analyses indicate conserved D3/D7a architecture across Oligostraca/Branchiopoda and higher variability with frequent expansions in Malacostraca.

**Conclusions:**

Our data extend RNA profile diversity to Oligostraca, refine fragment-size estimates with higher-resolution capillary electrophoresis, and link malacostracan profile heterogeneity to D7a and other expansions. We recommend rRNA-aware quality control for arthropod samples and targeted sequencing of poorly sampled lineages (*e.g.*, Mystacocarida, Cephalocarida, Remipedia) to resolve mechanisms and the phylogenetic distribution of the hidden break.

## Introduction

The hidden break is a post-transcriptional cleavage that removes a short fragment from 28S rRNA, dividing it into 28Sa and 28Sb with the size of each often approximating that of 18S. Such hidden breaks characterize many protostome invertebrates, 57% of all the non-arthropod protostomes listed by Natsidis et al. (2019: their Fig. 2B), and 93% of the listed arthropods. Electrophoretic RNA profiles in arthropods therefore often show a single intense peak resulting from a mixture of 18S and 28S (Ishikawa & Newburgh, 1972; Ware, Renkawitz & Gerbi, 1985; Winnebeck, Millar & Warman, 2010). This hides the 28S bands and causes traditional measures of the quality of RNA in extracted samples to classify high-quality RNA as subpar. That is, conventional RNA Integrity Number (RIN) scores tend to underestimate RNA quality in taxa with a hidden break (Winnebeck, Millar & Warman, 2010). Notably, the break is never a problem *in vivo* because the two 28S fragments remain hydrogen-bonded in living cells and ribosome architecture is preserved.

The cleaved segment varies among taxa (for example, 19 nt in the dipteran *Sciara coprophila* and 41 nt in *Artemia parthenogenetica*) and consistently maps to an AU-rich window within the D7a expansion segment (Ware, Renkawitz & Gerbi, 1985; Fujiwara & Ishikawa, 1986; Sun et al., 2012). Two candidate cleavage signals recur near the junction: a UAAU tetramer ∼10 nt upstream of the 5′ end of 28Sb (Fujiwara & Ishikawa, 1986) and a conserved CGAAAGGG motif (McCarthy, Dugon & Power, 2015). A broad survey showed that pancrustacean profiles can be complex (up to five peaks), not just one or two (DeLeo et al., 2018).

Pancrustacea comprises three superclasses (*i.e.,* Oligostraca, Multicrustacea and Allotriocarida) collectively representing a very large fraction of described animal diversity and widely used in phylogenomic syntheses (Oakley et al., 2013; Lozano-Fernández et al., 2019; Bernot et al., 2023). Here, we extend taxonomic coverage (including Oligostraca), refine rRNA sizing with capillary electrophoresis, and analyze 28S D-regions to relate observed profile classes to expansion-segment evolution (Ruiz-Linares, Hancock & Dover, 1991).

## Materials and Methods

### Sampling and preservation

We sampled live specimens from 12 species spanning Branchiopoda, Malacostraca, and Oligostraca in temporary ponds, saltmarshes, freshwater springs, and caves around Valencia (Spain). Specimens were euthanized by snap-freezing in microcentrifuge tubes to minimize pain, suffering, and distress. DNA/RNA Shield (ECOGEN, Barcelona) was added to stabilize RNA, and tissues were homogenized with sterile plastic pestles. Lysates were centrifuged to pellet debris, and 200 µL of supernatant was transferred to a new tube.

### RNA extraction and quality control

Total RNA was extracted with the Quick-RNA MagBead kit (Zymo Research, Irvine, CA, USA) following the manufacturer’s protocol and eluted in 25–50 µL nuclease-free water. Two microliters of each extract were quantified on a NanoDrop, applying a ≥15 ng/µL threshold for profiling. Qualified samples were run at SCSIE (University of Valencia) using the Agilent RNA kit on a 5200 Fragment Analyzer. Samples and RNA ladder were denatured at 70 °C for 2 min, cooled to 4 °C, and held on ice prior to loading (final mix: 2 µL sample + 22 µL RNA Diluent Marker, 15 nt).

**Table 1:** Estimated length of the 18S and 28S rRNA genes (complete or near-complete in GenBank) and the D3 and D7a regions across Pancrustacea. Lengths were directly estimated using Fragment Analyzer for extracted RNA or based on sequence data (complete or near-complete in GenBank).

**Order**	**Species**	**18S length**	**28S length**	**28Sa**	**28Sb**	**D3 position**	**D7a position**	**18S, 28S Genbank/ This study**
Podocopida (Ostracoda)	*Eucypris* sp. (partial)	1,874	3,387	1,817	1,587	919–1,032 (113)	1,817–1,910 (93)	AY457059 (18S) AB675007 (28S)
Podocopida (Ostracoda)	*Eucypris virens*	1,965 ± 11	3,930	1,965 ± 11	1,965 ± 11			This study
Podocopida (Ostracoda)	*Cyprideis torosa*	1,775 ± 73	3,550	1,775 ± 73	1,775 ± 73			This study
Podocopida (Ostracoda)	*Cyprideis torosa*	1,814	3,458	1,760	1,600	872–980 (108)	1,760–1,858 (98)	CAJPEW010005242.1 New annotation
Anostraca (Branchiopoda)	*Artemia franciscana*	1,800	3,253	1,722	1,553	836–929 (92)	1,722–1,821 (99)	XR_010618359 (18S) XR_010618360 (28S) New annotation
Anostraca (Branchiopoda)	*Artemia salina*	1,789 ± 26	3,446	1,723 ± 58	1,723 ± 58			This study
Anostraca (Branchiopoda)	*Branchipus schaefferi*	1,876 ± 20	3,752	1,876 ± 20	1,876 ± 20			This study
Notostraca (Branchiopoda)	*Triops* sp. (partial)	N/A	3,314	1,764	1,514	879–978 (99)	1,764–1,863 (99)	AY210844 (28S)
Notostraca (Branchiopoda)	*Triops cancriformis* (partial)	1,784	3,277	1,727	1,477	842–941 (99)	1,727–1,826 (99)	EU370422 (18S) AY744896 (28S)
Notostraca (Branchiopoda)	*Triops simplex*	1,919 ± 37	3,838	1,919 ± 37	1,919 ± 37			This study
Notostraca (Branchiopoda)	*Triops longicaudatus*	1,809	3,728	1,756	1,881	875–972 (97)	1,756–1,847 (91)	AF144219 (18S) JAGQDJ010000411.1 (28S) New annotation
Diplostraca (Branchiopoda)	*Daphnia magna*	2,341	3,775	1,871	1,975	925–1,052 (126)	1,871–2,106 (235)	AM490278 (18S) XR_006642556 (28S) New annotation
Diplostraca (Branchiopoda)	*Daphnia pulicaria* (partial)	2,294	3,964	1,869	2,164	923–1,056 (131)	1,869–2,247 (378)	XR_006919928 (18S) AF346514 (28S) New annotation
Isopoda (Malacostraca)	*Porcellio scaber* (partial)	3,192	4,548	2,163	2,130	986–1,317 (331)	2,163–2,418 (255)	AJ287062 (18S) EU914253 (28S)
Isopoda (Malacostraca)	*Lekanesphaera hookeri*	2,803 ± 131	4,437	2,059 ± 138	2,378 ± 161			This study
Isopoda (Malacostraca)	*Ceratothoa steindachneri*	N/A	3,809	1,897	1,623	940–1,111 (171)	1,897–2,186 (289)	qmCerStei3.1 (28S) New annotation
Isopoda (Malacostraca)	*Trachelipus rathkii*	3,333	4,882	1,873	2,234	738–1,048 (309)	1,873–2,648 (775)	JABUQX010117964.1 (18S) JABUQX010145190.1 (28S) New annotation
Isopoda (Malacostraca)	*Cirolanidae* sp.	3,008 ± 81	4,883	1,100 ± 60	3,783 ± 118			This study
Isopoda (Malacostraca)	*Asellus aquaticus*	2,080	3,343	1,357	1,813	244–563 (318)	1,357–1,530 (173)	OZ125847.1 New annotation
Amphipoda (Malacostraca)	*Gammarus roeselii*	2,239	4,691	2,023	2,172	736–1,188 (452)	2,023–2,519 (496)	SDVV010205854.1 (18S) SDVV010058929.1 (28S) New annotation
Amphipoda (Malacostraca)	*Haploginglymus* sp.	2,446	4,892	2,446	2,446			This study
Amphipoda (Malacostraca)	*Echinogammarus* sp.	2,439	4,220	2,110	2,110			This study
Amphipoda (Malacostraca)	*Riphidogammarus* sp.	2,395	4,198	2,099	2,099			This study
Amphipoda (Malacostraca)	*Sensonator valentiensis*	2,873	4,564	2,282	2,282			This study
Decapoda (Malacostraca)	*Eriocheir sinensis*	1,871	4,957	2,076	2,757	851–1,272 (421)	2,076–2,200 (123)	XR_007756995 (18S) XR_007735887 (28S) New annotation
Decapoda (Malacostraca)	*Halocaridina rubra*	1,855	4,088	1,914	1,899	1,005–1,123 (118)	1,914–2,189 (275)	JAXCGZ010021359.1 New annotation
Decapoda (Malacostraca)	*Dugastella valentina*	2,071 ± 133	4,632	1,903 ± 87	2,729 ± 169			This study
Hymenoptera (Hexapoda)	*Apis mellifera*	1,923	3,538	1,905	1,638	989-1,089 (100)	1,905-2,012 (107)	XR_003306453 (18S) XR_003306454 (28S) New annotation

### Peak assignment and comparative profiling

We analyzed RNA profiles for all species (Table 1). Peaks were assigned to 28S and 18S fragments by estimating gene lengths from BLAST matches of complete rRNA sequences from related species (almost all of them are related to our taxa at the suborder down to the genus level) against reference genomes (*e.g.*, *Asellus aquaticus, Trachelipus rathkii, Halocaridina rubra, Gammarus roeselii, Artemia franciscana, Triops longicaudatus, Cyprideis torosa*). Gene size was computed as end–start of the match, and peaks were assigned accordingly (full or partial gene). In R, we calculated the mean and SD of major-peak sizes per species and plotted boxplots (median and IQR) for representative species of each main group (*i.e.,* Branchiopoda, Malacostraca, and Oligostraca).

### Hidden-break validation (D7a)

To test whether the hidden break lies in D7a, we compared observed fragment sizes with the D7a position in reference species whose 28S is complete or near-complete in GenBank (*e.g.*, *Eucypris* sp.*, Cyprideis torosa, Artemia franciscana, Daphnia magna, Daphnia pulicaria, Triops cancriformis, Triops longicaudatus, Ceratothoa steindachneri, Porcellio scaber, Asellus aquaticus, Trachelipus rathkii, Gammarus roeselii, Eriocheir sinensis,* and *Halocaridina rubra*). We aligned annotated *Apis mellifera* 28S to these taxa with Muscle in BioEdit, inspected alignments and predicted/visualized secondary structures with RNAfold. The same workflow was applied to rRNA sequences obtained *via* BLAST when annotated references were unavailable.

## Results

**Figure 1:**
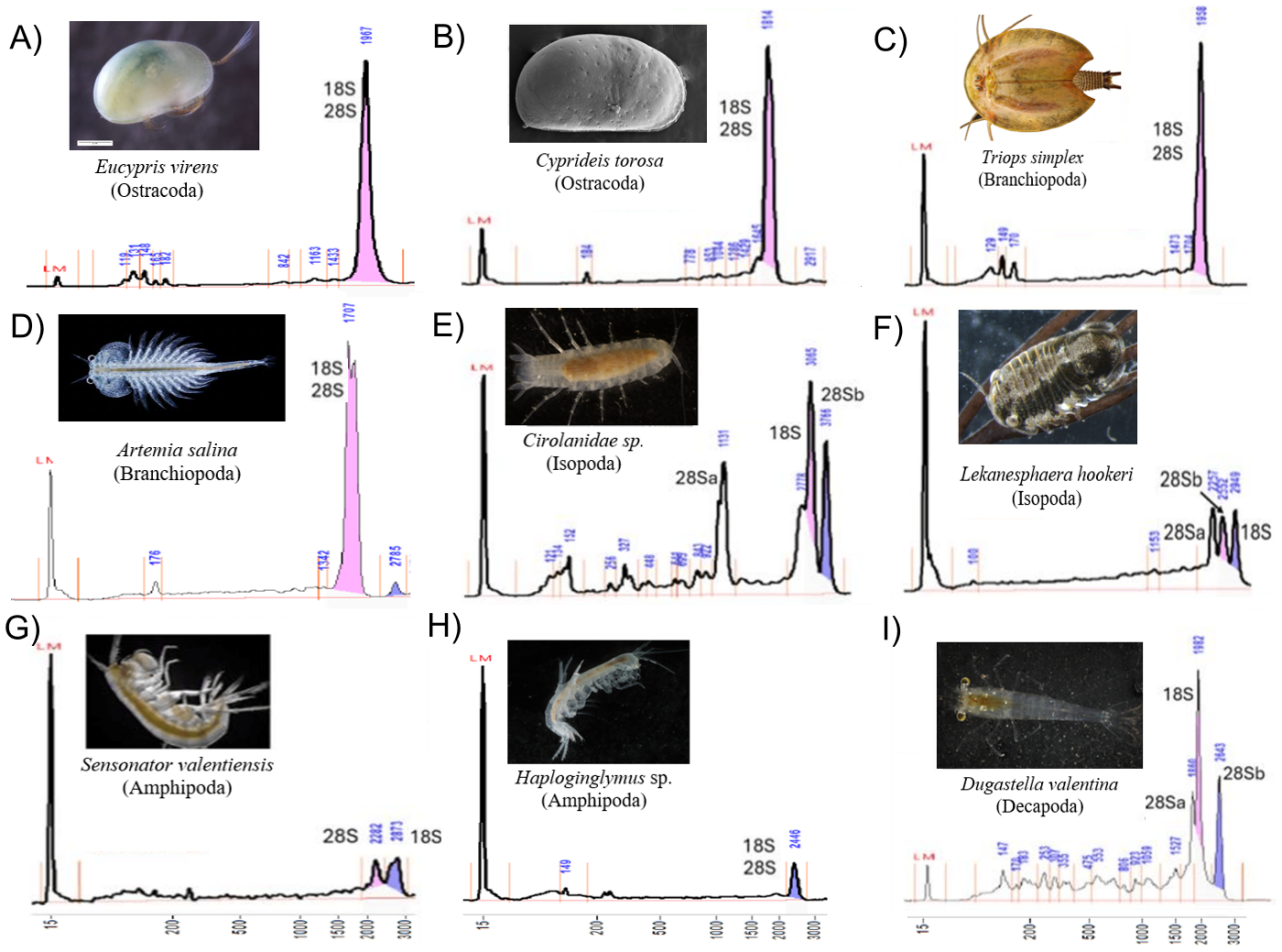
Diversity of Pancrustacea’s RNA profiles. Profiles differ markedly among Oligostraca (A–B), Branchiopoda (C–D) and Malacostraca (E–I) lineages. Details on peak sizes are available as Supplemental Information 2. Peak coloring follows Fragment Analyzer software by-default (pink and purple for the first and second tallest peaks). LM peak corresponds to the RNA Diluent Marker (15 nt).

Electropherograms varied by clade, from one to three dominant peaks. Oligostracan and branchiopod species showed the canonical single-peak pattern consistent with overlapping 18S, 28Sa and 28Sb (Figs. 1A–1D). Their 28S lengths ranged from ∼3,250–4,000 bp, and 18S from ∼1,700–2,300 bp (Table 1). In *Artemia salina*, two closely spaced peaks frequently merged into one measurement. Malacostracans were more variable (Figs. 1E–1I): most showed multiple peaks, with longer 28S (∼3,343–4,957 bp) and 18S (∼1,855–3,333 bp) than in the other pancrustacean clades. Isopods exhibited three peaks, consistent with a 28S split into unequal fragments, but they differed in which peak corresponded to 18S. For example, 18S forms the peak representing the longest fragment in *Lekanesphaera* (Fig. 1F) but it forms the peak for the *second*-longest fragment in Cirolanidae (Fig. 1E). Amphipods generally showed two peaks that were consistent with an 18S peak plus the 28S peak (except *Haploginglymus* sp., which had a single peak that contained 28Sa, 28Sb, and 18S). Overall, the isopods and amphipods have relatively large rRNA molecules with their 18S larger than either their 28Sa or 28Sb. Decapods resembled the cirolanid isopod in showing three peaks, with 18S as the middle peak (Fig. 1, Fig. S1). Also, decapods and isopods were the only clades whose 28Sa and 28Sb markedly differed from one another in length (see the Supplemental Information 2).

Across taxa with a single peak, co-migration of similarly sized fragments reduces sizing precision, as expected. Occasionally, a very small 28S full-length peak was visible, presumably from incompletely denatured molecules. Fragment lengths measured by gel electrophoresis mostly match those from the sequenced rRNA reported in the referenced literature (Table 1). For example, in *Cyprideis torosa* the sequence in GenBank (CAJPEW010005242.1) documents D7a at positions 1,760–1,858 bp, which nicely matches the break calculated from our gels at ∼1,775 ± 73 bp (*i.e.*, the two different measurements of 28Sa match well). In addition, our gel-derived 28Sa-lengths for *Eucypris, Artemia*, and *Triops* match those from sequences of the congeneric animals within 6%, a close fit: 1,965 *versus* 1,860 bp for *Eucypris*, 1,723 *versus* 1,772 for *Artemia*, and 1,919 *versus* 1,800 for *Triops*. Although the amphipod and isopod species we used for our gels were not so closely related to the referenced taxa, the latter still proved to be valid proxies. That is, the average 28Sa length for our four amphipods (2,230 bp) was remarkably close to that of amphipod *Gammarus* from the literature (2,270 bp); and the length of 28Sa in our isopod *Lekanesphaera* (2,059 bp) was close to the average for the four isopods from the literature (2,000 bp). All these close matches justify our choice to use proxies instead of sequencing the rRNA of our own taxa. However, this approach of relying so heavily on gel-based lengths was not perfect, given that it estimated the length of 28Sa in Cirolanidae species at 1,100 bp. That is too small and unlikely to be correct because anything shorter than 1,700 bp is seldom seen in eukaryotes.

**Figure 2:**
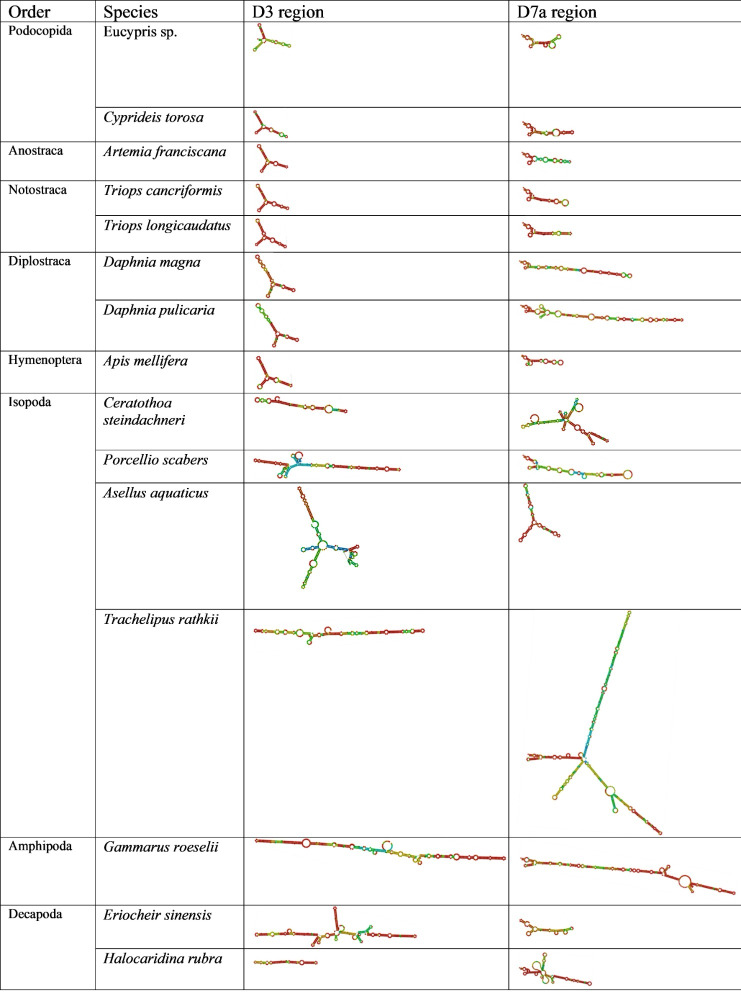
Secondary structure of the D3 and D7a regions of 28S genes obtained from GenBank. The colors indicate the base-pair probabilities, with blue being the least accurate and red being the most accurate. For unpaired regions the color denotes the probability of being unpaired.

Across orders, D3 length and structure were similar in Ostracoda, Branchiopoda and Insecta, but longer in some Malacostraca. Similarly, D7a variability was greatest in Malacostraca (notably Isopoda) with notable elongation in Diplostraca (Fig. 2). The CGAAAGGG motif was present at the end of D7a in all species examined except *Ceratothoa steindachneri* (73 bp upstream); a second short motif (CCTAAG) occurred at the D7a start in all species; and UAAU was frequent in Isopoda and Amphipoda.

## Discussion

Our most important finding is that Oligostraca shares the canonical single-peak profile with most branchiopods, whereas Malacostraca exhibits markedly greater diversity linked to unequal enlargements of expansion segments in 28S, including D3 and D7a. This extends the pancrustacean survey (DeLeo et al., 2018) by adding Oligostraca and improving sizing precision with capillary electrophoresis.

The sizes of 28Sa/b fragments track the mapped position of D7a expansions, consistent with classic mapping that places the excision in an AU-rich D7a loop and identifies short sequence/structural cues (Fujiwara & Ishikawa, 1986; Ware, Renkawitz & Gerbi, 1985; Sun et al., 2012). Expansion segments are not neutral: growing evidence links them to ribosome dynamics and translational regulation (Rauscher & Polacek, 2024) or to preserving the AT/GC balance in the functional core of 28S rRNA (Mallatt & Chittenden, 2014). Conserved base-pairing *via* compensatory substitutions suggests selection to preserve helical stems despite length change (Ruiz-Linares, Hancock & Dover, 1991).

Comparative evidence indicates that 28S expansion segments evolve under strong structural constraints despite wide length variation. Mallatt, Craig & Yoder (2012) showed that metazoan D-regions retain conserved secondary-structure cores maintained by compensatory substitutions, and our results extend this framework to Pancrustacea: D3 is relatively conserved across Oligostraca and Branchiopoda, whereas it shows lineage-specific expansions in some Malacostraca. In arthropods, D3 functions as a structurally constrained yet length-variable surface appendage of the LSU, often expanding *via* simple-sequence accretion but preserving its helical core (Whiting et al., 1997; Gillespie et al., 2004; Gillespie, Yoder & Wharton, 2005). Structure-guided alignments of D3 have strong phylogenetic utility, and by analogy to other eukaryotic expansion segments, D3 and D7a may act as tunable scaffolds that modulate ribosome-factor or ribosome-RNA interactions without disturbing the catalytic core (Ruiz-Linares, Hancock & Dover, 1991; Mallatt, Craig & Yoder, 2012; Rauscher & Polacek, 2024).

Our clade-specific patterns generally agree with DeLeo et al. (2018), but we detect additional bands in some amphipods and decapods, likely due to the higher resolution of the Fragment Analyzer. A strength of this study is the integration of electropherograms with mapped D-region coordinates across lineages; it indicates that the pherograms do contain both 28Sa and 28Sb peaks, even when hidden by the 18S peak. Most importantly, it confirms that RIN-based Quality Control (QC) is unreliable when a hidden break collapses the 28S band; rRNA-aware QC should be preferred (Winnebeck, Millar & Warman, 2010). Limitations of the study include modest species sampling and reliance on related species for genomic inferences about their taxa. The ambiguous shoulder near the cirolanid 18S peak (Fig. 1E) could reflect co-migration or secondary structure; targeted Northern blots or RNase mapping would resolve this with the help of a full sequencing of the cirolanid rRNA genes in the future.

## Conclusions

We expand RNA profile diversity to Oligostraca, amphipods, and isopods, and strengthen the link between malacostracan profile heterogeneity, 28S expansion segments, and the hidden break in D7a. We recommend rRNA-aware quality control for arthropods and targeted sequencing of undersampled lineages (Mystacocarida, Cephalocarida, Remipedia) to test hidden-break generality, identify cleavage cues, and integrate profile classes with rDNA architecture. Speculative but testable predictions include a causal role for local stem-loop motifs in D7a processing and lineage-specific mobile-element associations near D7/D8.

## Supplemental Information

10.7717/peerj.20693/supp-1 Supplemental Information 1 Intraspecies size variation (base pairs) of the peaks of the Fragment Analyzer RNA profiles in different Pancrustacea

10.7717/peerj.20693/supp-2 Supplemental Information 2 Length of the 18S and 28S rRNA genes across Pancrustacea Fragment Analyzer measurements (raw data) were carried out at the SCSIE facilities soon after the extraction.

## Additional Information and Declarations

### Data Availability

The following information was supplied regarding data availability: The raw measurements are available in the Supplemental File.
