# Supplementary figures and images for: Hidden-break diversity in pancrustacean rRNA profiles
Source: PeerJ. 2026 Feb 3;14:e20693. doi: 10.7717/peerj.20693 (PMC12880105; doi:10.7717/peerj.20693)

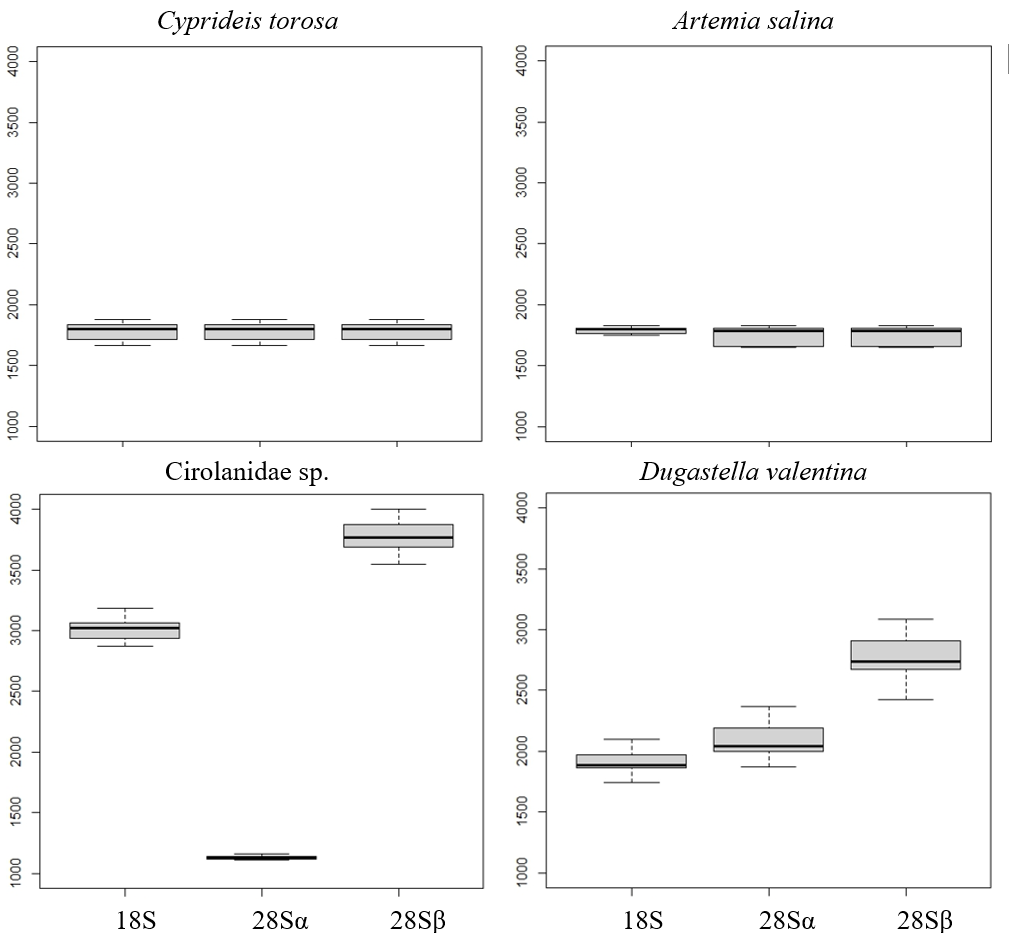

Supplement: Supplemental Information 1 [file peerj-14-20693-s001.png]
